# Transcriptional analysis of multiple ovarian cancer cohorts reveals prognostic and immunomodulatory consequences of ERV expression

**DOI:** 10.1136/jitc-2020-001519

**Published:** 2021-01-12

**Authors:** Marina Natoli, John Gallon, Haonan Lu, Ala Amgheib, David J Pinato, Francesco A Mauri, Teresa Marafioti, Ayse U Akarca, Ines Ullmo, Jacey Ip, Eric O Aboagye, Robert Brown, Anastasios Karadimitris, Sadaf Ghaem-Maghami

**Affiliations:** 1 Department of Surgery and Cancer, Imperial College London, London, UK; 2 Department of Biomedicine, University Hospital Basel, Basel, Switzerland; 3 Department of Pathology, University College London Cancer Institute, London, UK; 4 Department of Pathology, Institute of Cancer Research, London, UK; 5 Department of Immunology and Inflammation, Imperial College London, London, UK

**Keywords:** biomarkers, tumor, computational biology, interferon inducers

## Abstract

**Background:**

Endogenous retroviruses (ERVs) play a role in a variety of biological processes, including embryogenesis and cancer. DNA methyltransferase inhibitors (DNMTi)-induced ERV expression triggers interferon responses in ovarian cancer cells via the viral sensing machinery. Baseline expression of ERVs also occurs in cancer cells, though this process is poorly understood and previously unexplored in epithelial ovarian cancer (EOC). Here, the prognostic and immunomodulatory consequences of baseline ERV expression was assessed in EOC.

**Methods:**

ERV expression was assessed using EOC transcriptional data from The Cancer Genome Atlas (TCGA) and from an independent cohort (Hammersmith Hospital, HH), as well as from untreated or DNMTi-treated EOC cell lines. Least absolute shrinkage and selection operator (LASSO) logistic regression defined an ERV expression score to predict patient prognosis. Immunohistochemistry (IHC) was conducted on the HH cohort. Combination of DNMTi treatment with γδ T cells was tested *in vitro*, using EOC cell lines and patient-derived tumor cells.

**Results:**

ERV expression was found to define clinically relevant subsets of EOC patients. An ERV prognostic score was successfully generated in TCGA and validated in the independent cohort. In EOC patients from this cohort, a high ERV score was associated with better survival (log-rank p=0.0009) and correlated with infiltration of CD8+PD1+T cells (r=0.46, p=0.0001). In the TCGA dataset, a higher ERV score was found in BRCA1/2 mutant tumors, compared to wild type (p=0.015), while a lower ERV score was found in CCNE1 amplified tumors, compared to wild type (p=0.019). *In vitro*, baseline ERV expression dictates the level of ERV induction in response to DNMTi. Manipulation of an ERV expression threshold by DNMTi resulted in improved EOC cell killing by cytotoxic immune cells.

**Conclusions:**

These findings uncover the potential for baseline ERV expression to robustly inform EOC patient prognosis, influence tumor immune infiltration and affect antitumor immunity.

## Background

About 40% of the human genome consists of repetitive sequences. Among these, endogenous retroviruses (ERVs) are a class of transposable elements (TE) that derive from ancient exogenous retroviral infections resulting in incorporation of the viral genome into the host.^1^

Though ERVs are usually silenced by heavy DNA and histone methylation, ERV transcripts seem to play a role in early mammalian development, with high transcriptional activity of distinct ERV families being observed in human embryos during pre-implantation development^2^ while, in cancer, aberrant expression of TE has been hypothesized to drive tumorigenic mutations.^3^

DNA methyltransferase 1-deficient mice develop T cell leukemia in the absence of functional Toll-Like Receptors, partly via ERV hypomethylation and deregulation,^4^ while in human colon cancer samples, RNA *in situ* hybridisation demonstrated a correlation between HERV-H expression and localisation of suppressive infiltrating Tregs.^5^

High levels of expression of specific ERVs were identified in clear cell renal cell carcinoma, breast, colon, and head and neck cancers from TCGA, and correlated with increased immune infiltration, particularly a high CD8+ T cell fraction as well as checkpoint pathway upregulation.^6^

Interestingly, treatment with the DNA methyltransferase inhibitor (DNMTi) decitabine can induce transcription of ERVs into double-stranded RNA (dsRNA) and mimic a viral infection, triggering an interferon (IFN) response.^7 8^

This literature highlights a role of TEs, including ERVs, in cancer and immunity which is not fully clarified or understood, with their expression being linked to tumor initiation and evolution, as well as stimulation of antitumoral innate immunity and recruitment of both Tregs and cytotoxic T lymphocytes to the tumor microenvironment.

Importantly, a strong correlation exists between the presence of intratumoral T cells and improved clinical outcome in advanced ovarian carcinomas.^9^ Epithelial ovarian cancer (EOC) is usually diagnosed at an advanced stage and carries a poor prognosis and it is therefore crucial to find new tools to stratify patients and design effective therapeutic interventions.

Pretreatment with epigenetic therapy has emerged as a potential strategy to stimulate immunologically cold tumors, including EOC, toward a less immunosuppressive and immune ‘evasive’ phenotype.^10 11^

Here, for the first time, we investigated the significance of ERV expression at basal level in high-grade serous ovarian tumors and again in the context of DNMTi treatment of EOC cell lines. Our findings demonstrate the influence of baseline ERV expression on patient survival and on immune cell infiltration into EOC tumors and confirm the potential for manipulation of an ERV expression threshold by DNMTi treatment.

## Results

### Baseline ERV expression defines subsets of EOC patients and informs patient survival

Given the dual role of ERV expression in cancer, and the importance of immune infiltration for OC prognosis, we first investigated baseline ERV expression in ovarian tumor expression data from TCGA.

**Figure 1:**
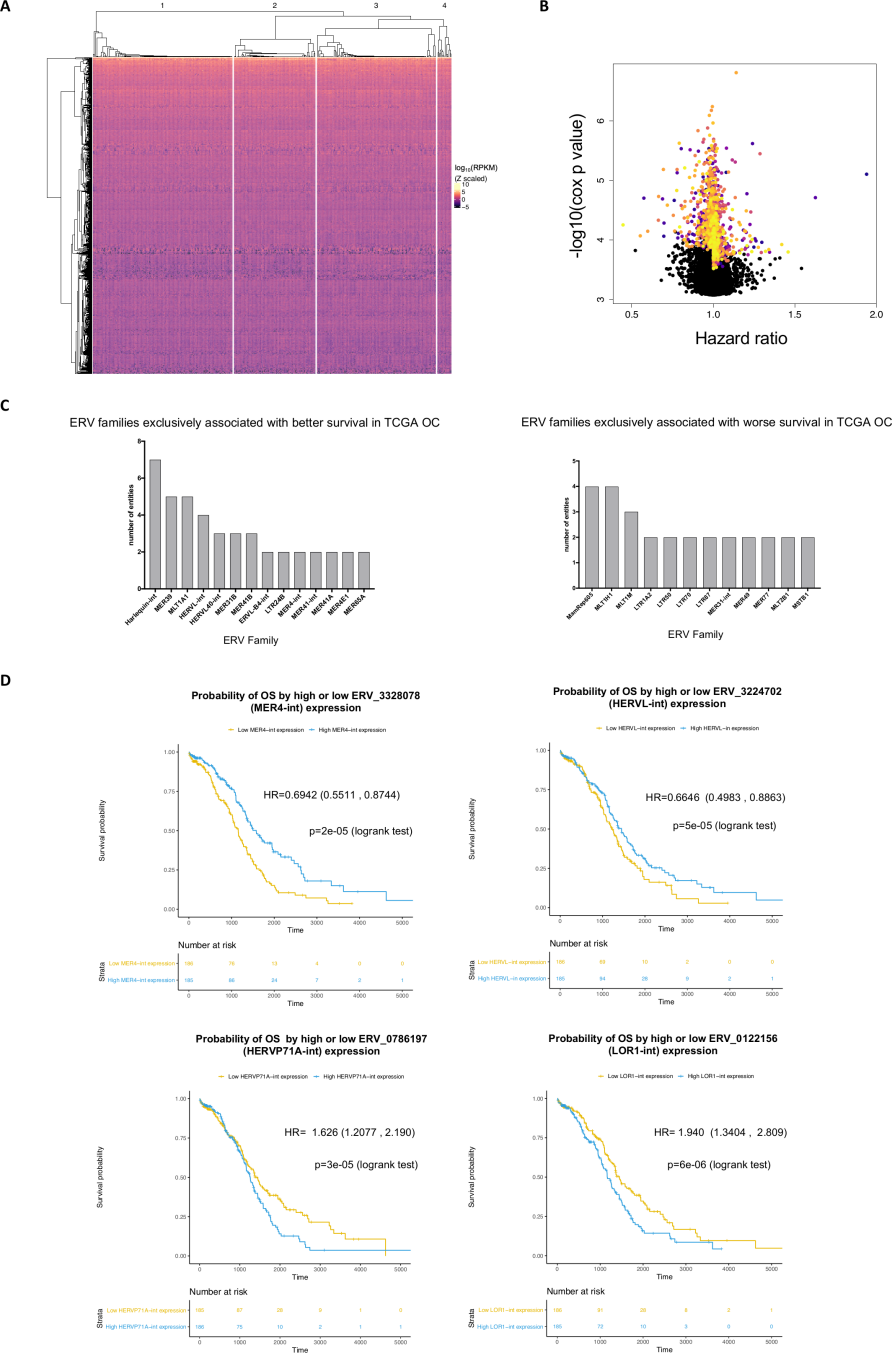
Baseline ERV expression defines subsets of OC patients and informs patient survival. (A) Heatmap showing the ERVs (n=1000) used for consensus clustering analysis and the TCGA OC samples (n=373), grouped using the dendrogram resulting from k=4 clustering. The colors show the z-scaled log10 of the ERV expression and are defined in the color scale. (B) Volcano plot showing the calculated multivariate Cox regression models between each ERV repeat’s expression and OS in TCGA OC dataset. HRs were plotted against the negative log10 of the adjusted cox p value for the 25 207 ERV repeats found expressed in TCGA OC samples. Multivariable Cox models were adjusted for age, stage, grade, histology and residual disease, using ERV expression values as continuous variables. Significant HRs (p<0.05) are colored. HR <1 indicates association between ERV expression and improved OS (n ERVs=632 for Cox p<0.05); HR >1 indicates association between ERV expression and worse OS (n ERVs=1187, Cox p<0.05). (C) ERV families with more than one ERV repeat, exclusively associated with better (left) or worse (right) survival in TCGA OC dataset, annotated with name of the family and number of entities (ie, repeats). (D) Kaplan-Meier plots of OS according to above median (high) or below median (low) expression (ie, RPKM) of selected ERV repeats. The ERV family is indicated in brackets. The HR was estimated by a multivariable Cox model adjusted for age, stage, grade, histology and residual disease. The CI is indicated, in brackets. ERV, endogenous retrovirus; OC, ovarian cancer.

A total of 25 207 ERV repeats were found expressed in all primary OC samples (n=373) and consensus clustering analysis identified four main clusters, defined by ERV expression. This indicates that different patterns of ERV expression define subgroups of EOC patients (figure 1A and online supplemental figure S1).

10.1136/jitc-2020-001519.supp1 Supplementary data

10.1136/jitc-2020-001519.supp2 Supplementary data

Next, we generated multivariable Cox models—adjusted for age, stage, grade and residual disease—to determine whether the expression of each single ERV repeat in the TCGA dataset (n=25 207) was associated with overall survival (OS). For each ERV, samples with complete clinical data (n=328) were allocated to groups (high or low) using the ERV repeat’s median expression level as cut-off. Of the 25 207 ERVs tested, 632 had a favorable association with OS (Cox p<0.05) and 1187 an unfavorable association (Cox p<0.05) (figure 1B).

Interestingly, some ERV families, that is groups of ERV repeats with the same sequence but at different genomic loci, were associated with both favorable and unfavorable OS (online supplemental table S1), suggesting that the repeat location, rather than the family or sequence, may have a predominant role in affecting OS.

10.1136/jitc-2020-001519.supp3 Supplementary data

The ERV families that were exclusively associated with either favorable (n=58) or unfavorable OS were identified (n=76) and those with more than one ERV repeat associated with OS, are shown in figure 1C. The ERV repeat ERV_3328078 belonging to the ERV family MER4-int was found to have the lowest HR, ie, high expression of this ERV was significantly associated with the highest survival advantage (HR 0.69, p=0.001). Similarly, ERV repeat ERV_3224702 (HERVL-int family) presented the second lowest HR (figure 1D, top). Instead, ERV repeats ERV_0122156 (LOR1-int family) and ERV_0786197 (HERVP71A-int family) presented the top and second highest HRs, indicating that patients presenting low (ie, below median) expression of these repeats are more likely to survive for longer (figure 1D, bottom).

### An ERV expression score predicts good prognosis in EOC patients

**Figure 2:**
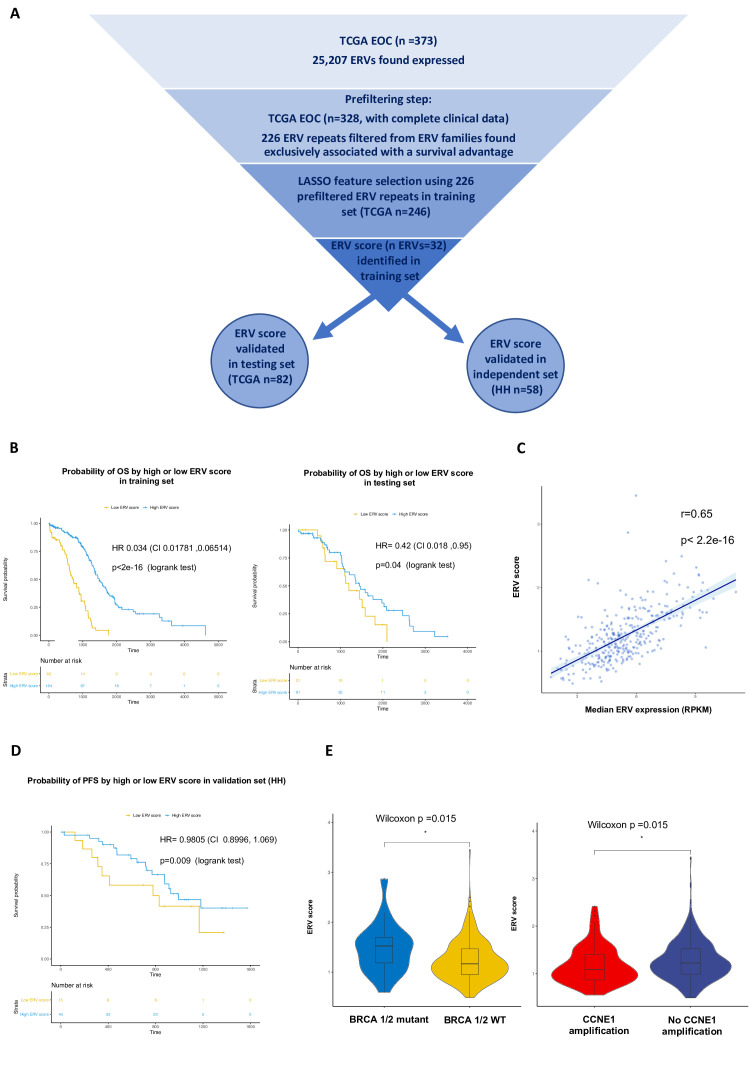
An ERV expression score predicts good prognosis in OC patients. (A) Schematic representation of the steps and datasets used in generating the ERV prognostic score. (B) Overall survival (OS) of OC patients by high (above first quartile) or low (below first quartile) ERV prognostic score in the training (left; n samples=246) and testing (right; n samples=82) sets from TCGA. The HR was estimated by a multivariable Cox model adjusted for age, stage, grade, histology and residual disease (log-rank p values as well as CI are indicated). (C) Pearson’s product-moment correlation between the median ERV RPKM of the 32 ERV components of the ERV prognostic score and the prognostic score in OC TCGA samples (n=328); the shaded area indicates the confidence interval (0.58, 0.70) (D) Progression-free survival (PFS) of high-grade serous ovarian cancer (HGSOC) patients by high (above first quartile) or low (below first quartile) ERV prognostic score in Hammersmith Hospital (HH) validation dataset (n samples=58). The HR was estimated by a multivariable Cox model adjusted for age, stage, grade, histology and residual disease (log-rank p values are indicated). The CI is indicated, in brackets. (E) Left: boxplots showing the ERV score in BRCA 1/2 mutant tumors (n=21; including all types of somatic mutations except silent mutations), compared with wild-type tumors (n=307) from the TCGA dataset. Right: boxplots showing the ERV score in tumors with CCNE1 amplification (n=101), compared with tumors without CCNE1 amplification (n=227) from the TCGA dataset. P values were obtained using the Wilcoxon rank-sum test with continuity correction. EOC, epithelial ovarian cancer; ERV, endogenous retrovirus; LASSO, least absolute shrinkage and selection operator; OC, ovarian cancer.

A total of 226 ERV repeats were found to be significantly associated exclusively with an improved OS and further filtered using least absolute shrinkage and selection operator (LASSO^12^) to compute a prognostic score. Figure 2A shows a schematic representation of the steps and datasets used in developing the ERV score.

We first generated the model on a training set, consisting of 75% of the EOC TCGA samples with complete clinical data (n=246). Features (ERVs) were selected by a penalisation system, and weights were calculated for filtered features. The weighted sums of 32 selected ERVs resulted in a numerical score for each TCGA OC sample analyzed, which was named ERV score.

The 32 ERVs were annotated with ERV family and LASSO coefficients (online supplemental table S2). Online supplemental figure S2 shows each feature’s coefficient against the calculated LASSO parameter lambda and the optimal lambda value, indicating optimal number of features to be combined into the predictor score, obtained by 10-fold cross-validation using cv.glmnet within the glmnet package in R.

10.1136/jitc-2020-001519.supp4 Supplementary data

10.1136/jitc-2020-001519.supp5 Supplementary data

Multivariable Cox proportional hazards models, adjusted for age, stage, grade and residual disease, showed a significant difference in OS (figure 2B left) and progression-free survival (PFS) (online supplemental figure S3A) depending on a high (above first quartile) or low (below first quartile) ERV prognostic score. Figure 2B (left) shows the Kaplan-Meier survival curve for EOC patients in the training set (n=246), illustrating improved OS for patients with high (ie, above threshold) ERV prognostic score (log rank p<2e-16, HR=0.03405, 95% CI 0.0178 to 0.06513).

10.1136/jitc-2020-001519.supp6 Supplementary data

Next we validated the model in a testing set, consisting of the remaining 25%°C TCGA samples with complete survival data (n=82). In the testing set, similarly as in the training set, improved OS was significantly associated with a high ERV prognostic score (log rank p=0.04, HR 0.4239, 95% CI 0.1878 to 0.9567). The Kaplan-Meier plot for the testing set is shown in figure 2B (right). A similar effect was observed when calculating PFS on the testing test (online supplemental figure S3B).

In order to better interpret their biological significance, the ERV prognostic scores for each sample in TCGA (both training and testing sets) were correlated with the median ERV expression values of the 32 LASSO selected features. There was a Pearson’s product-moment correlation of 0.65 (p<2.2e-16), suggesting that higher levels of expression of the selected 32 ERVs may be associated with improved survival (figure 2C).

Importantly, the ERV prognostic score was successfully validated on an independent dataset, consisting of 58 samples from high-grade serous ovarian cancer (HGSOC) patients from Hammersmith Hospital London (HH dataset). The ERV score was calculated here by applying the previously generated LASSO weights to the expression of 23 ERV features shared by the TCGA and HH datasets. A high ERV score was significantly associated with improved PFS in the HH samples (log rank p=0.009, Cox model adjusted as above; figure 2D). It was not possible to calculate OS for these samples as the clinical information was too recent.

Given the impact that BRCA1/2 aberrations and CCNE1 amplification have on HGSOC prognosis,^13 14^ we next investigated whether there may be any association between the ERV score and these aberrations in the TCGA dataset. Strikingly, the ERV score was found to be significantly higher (Wilcoxon p=0.015) in BRCA 1/2 mutant tumors (n=21; including all types of somatic mutations except silent mutations), compared to wild-type tumors (n=307) and significantly lower (Wilcoxon p=0.019) in tumors with CCNE1 amplification (n=101), compared to tumors without CCNE1 amplification (n=227; figure 2E). Due to the fact that BRCA mutant tumors present better patient prognosis,^13 14^ while tumors with CCNE1 amplification present worse patient prognosis,^14 15^ these data support the prognostic value of the ERV score, with a high ERV score being associated with improved survival in HGSOC.

### The ERV score correlates with infiltration of effector immune cells in EOC

In order to investigate whether higher baseline ERV expression may affect immune cell infiltration of ovarian tumors, we calculated Pearson’s correlation coefficients between the expression of each of the 25 207 ERV repeats from the OC TCGA analysis and the expression of genes for T cell markers CD8, CD4, CD25, and activated or exhausted T cell markers LAG3 and PD-1, within the same samples.

**Figure 3:**
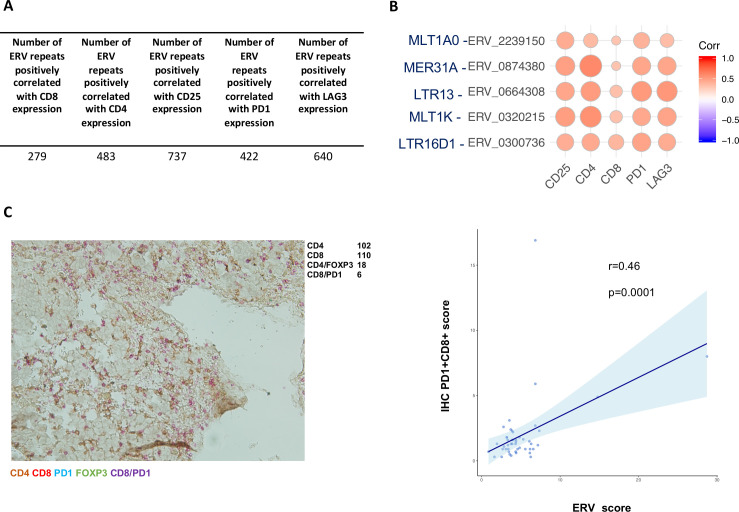
Immunomodulatory consequences of ERV expression in OC. (A) Summary of significant and positive (FDR adjusted p<0.05, r>0) Pearson’s correlations between the expression of ERV repeats and selected immune genes in TCGA OC samples with complete clinical data (n=328). (B) Correlation matrix of ERV repeats and T lymphocytes surface markers. Pearson’s product-moment correlations were calculated between each ERV repeat analyzed in TCGA OC dataset (n=25 207) and immune genes of interest CD25, CD4, CD8, PD1 and LAG3. Significant correlations (FDR adjusted p<0.05) were filtered by correlation coefficient (cut-off r>0.3) and only non-intragenic ERVs were retained. The color scale indicates the correlation coefficient and the size of the dot indicates the p value. (C) Correlation between ERV prognostic score and multiplex IHC CD8+PD1+scores in HH samples (n=47). A representative immune-rich sample from the HH cohort, stained by multiple IHC for CD4, CD8, PD1 and FOXP3 is shown on the left: colors are indicated in the legend (bottom) as well as representative scoring (top right). The CD8+PD1+double positive cells were scored and normalized by the total number of immune cells, generating an IHC PD1+CD8+score for each sample (n=47). The IHC PD1+CD8+score was correlated to the ERV score and plotted in R (right). ERV, endogenous retrovirus; HH, Hammersmith hospital; IHC, immunohistochemistry; OC, ovarian cancer.

Figure 3A shows the number of significantly positively correlated ERV repeats (false discovery rate (FDR) adjusted p<0.05, correlation coefficient r>0) for each of the immune genes of interest.

Five ERV repeats were found to be strongly correlated with all the immune genes of interest (figure 3B), indicating that these five ERVs may be translated into immunogenic antigens and attract effector T cells to the tumors. High individual expression of each of these five ERVs was also found to be associated with better survival in the TCGA dataset, though their prognostic value was limited compared to the combined ERV score (online supplemental figure S4).

10.1136/jitc-2020-001519.supp7 Supplementary data

Furthermore, significant positive correlations (p<0.05, r>0) were found between the ERV score and the expression of PD-1 (*Pdcd1*) and LAG3 (*Lag3*) (online supplemental figure S5, top), both normally found expressed on activated or exhausted T lymphocytes, in the EOC TCGA samples (n=328).

10.1136/jitc-2020-001519.supp8 Supplementary data

Moreover, the ERV score was found significantly positively correlated (p=0.01, r=0.14; online supplemental figure S5, bottom left) with the expression of the gene for viral recognition protein RIG-I (*Ddx58*), within the TCGA EOC samples (n=328), indicating that a high ERV score may result in higher expression of viral response genes. Similarly, a significant positive correlation was found between the ERV score and the expression of IFNβ (*Ifnb1;* p=0.03, r=0.11; online supplemental figure S5, bottom right). Since these associations did not present a strong correlation coefficient and in order to better validate the biological significance of the ERV score, we conducted multiplex IHC, staining for common markers of tumor infiltrating lymphocytes (TILs), on EOC samples from the HH dataset (n=47). Figure 3C (left) shows a representative immune-enriched EOC sample. Strikingly, a significant positive correlation (r=0.46, p=0.0001) was found between the ERV score and expression of CD8+PD1+ double positive cytotoxic T cells (figure 3C, right), strongly suggesting that a higher expression of these ERVs may increase immunogenicity and therefore recruitment or activation of effector immune cells.

### Baseline ERV expression in HGSOC cell lines

**Figure 4:**
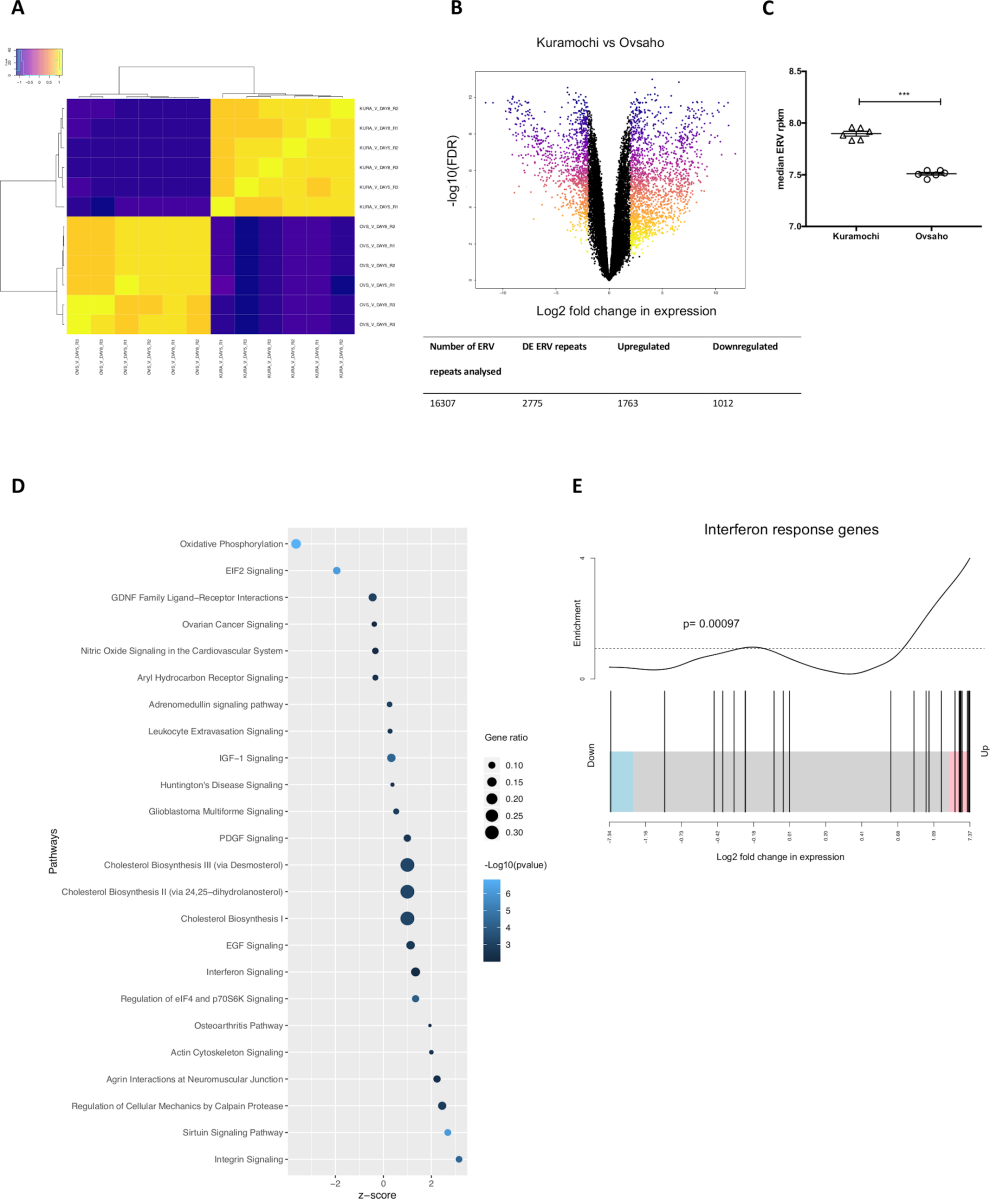
Baseline ERV expression in HGSOC cell lines. (A) Spearman’s correlation coefficients were calculated between experimental replicates in Kuramochi and Ovsaho cell lines for all ERV repeats analyzed. The dendrograms were generated by unsupervised hierarchical clustering and show the relationship between samples. The colors in each sample are indicative of the correlation coefficient and are defined in the color scale. (KURA=Kuramochi; OVS=Ovsaho; V=vehicle; DAY5=early timepoint; DAY8=late timepoint; R=replicate). (B) Top: Volcano plot showing the log2 fold change in expression against the –log10 FDR adjusted p value for each ERV repeat analyzed in vehicle-treated Kuramochi or Ovsaho HGSOC cell lines. Significantly DE ERV repeats (absolute logFC >2 and FDR adjusted p<0.05) are colored. Bottom: Summary of total number of ERV repeats analyzed in the Kuramochi versus Ovsaho comparison, including number and direction of change of each DE ERV repeat. (C) Median ERV expression in baseline Kuramochi and Ovsaho HGSOC cell lines. Median of RPKM values from all ERV repeats analyzed in each vehicle-treated sample for Kuramochi and Ovsaho. The median value for each sample is shown together with the mean±SEM (***p<0.001, t-test). (D) IPA was used to identify pathways positively or negatively regulated in Kuramochi compared with Ovsaho. A p value threshold of 0.01 was applied. The pathways were identified in IPA and visualized in R, annotated with negative log10 p value (blue color scale), gene ratio (number of DE genes in each pathway/total genes in the pathway; defined by the size of dot) and IPA-calculated activation z-score (indicative of upregulation or downregulation of genes; x axis). (E) Enrichment for interferon response genes in HGSOC cell line Kuramochi compared with Ovsaho. Mean-rank gene set tests were conducted to assess whether the genes from the cell lines’ analysis were highly ranked relative to an interferon response gene list, in terms of their logFC. P value was obtained from a Wilcoxon test. Each black line represents a gene in the interferon gene list, obtained from the Molecular Signatures Database (down=downregulated, up=upregulated). DE, differentially expressed; ERV, endogenous retrovirus; IPA, ingenuity pathway analysis.

In an effort to better understand the significance of baseline ERV expression in EOC and how this can be manipulated, we conducted RNA-sequencing of the Kuramochi and Ovsaho HGSOC cell lines. As expected, we found a clear separation between the cell lines, based on expression of all ERVs. Differentially expressed (DE) ERV repeats between the two cell lines (absolute log2FC>±2; FDR adjusted p<0.05) were then identified; 2775 DE ERV repeats were found to be DE (figure 4A); 1763 ERV repeats were upregulated in Kuramochi, compared with Ovsaho, while 1012 were downregulated (figure 4B).

Interestingly, the median ERV expression values, a surrogate measure of overall ERV expression, were significantly higher in the Kuramochi samples, compared with Ovsaho (figure 4C). When the gene expression profiles of the two cell lines were compared, genes for viral sensor protein RIG-I (ie, *Ddx58,* logFC 5.421 FDR adjusted p value 1.53–12) and MDA5 (ie, *Ifih1*, logFC 2.546, FDR adjusted p 2.84–08) were found upregulated in Kuramochi cells, compared with Ovsaho cells. Accordingly, ingenuity pathway analysis (IPA) revealed an enrichment for IFN signaling in Kuramochi cells, compared with Ovsaho cells (figure 4D). This enrichment was further confirmed by testing a specific IFN response gene list from the Molecular Signatures Database^16^ against all the genes in the analysis (figure 4E).

Altogether these data demonstrate the existence of distinct patterns of ERV expression in different HGSOC cell lines and confirms that a higher spontaneous expression of ERVs may determine increased expression of genes for antiviral mediators RIG-I and MDA5 and consequential IFN type I induction.

### Baseline ERV expression dictates magnitude of response to DNMTi and immune cell combination treatment in HGSOC cell lines

Viral mimicry via induced expression of ERVs has been described as a key consequence of epigenetic modification in cancer cells.^7 8^ Here, for the first time, genome-wide changes in ERV expression were investigated following 1 µM guadecitabine—a DNMTi—treatment of Kuramochi and Ovsaho cell lines.

As expected, treatment with guadecitabine resulted in a significant dose-dependent decrease in global DNA methylation, as measured by bisulfite pyrosequencing of Long Interspersed Nuclear Element-1, as a surrogate measure of global DNA methylation (online supplemental figure S6).

10.1136/jitc-2020-001519.supp9 Supplementary data

**Figure 5:**
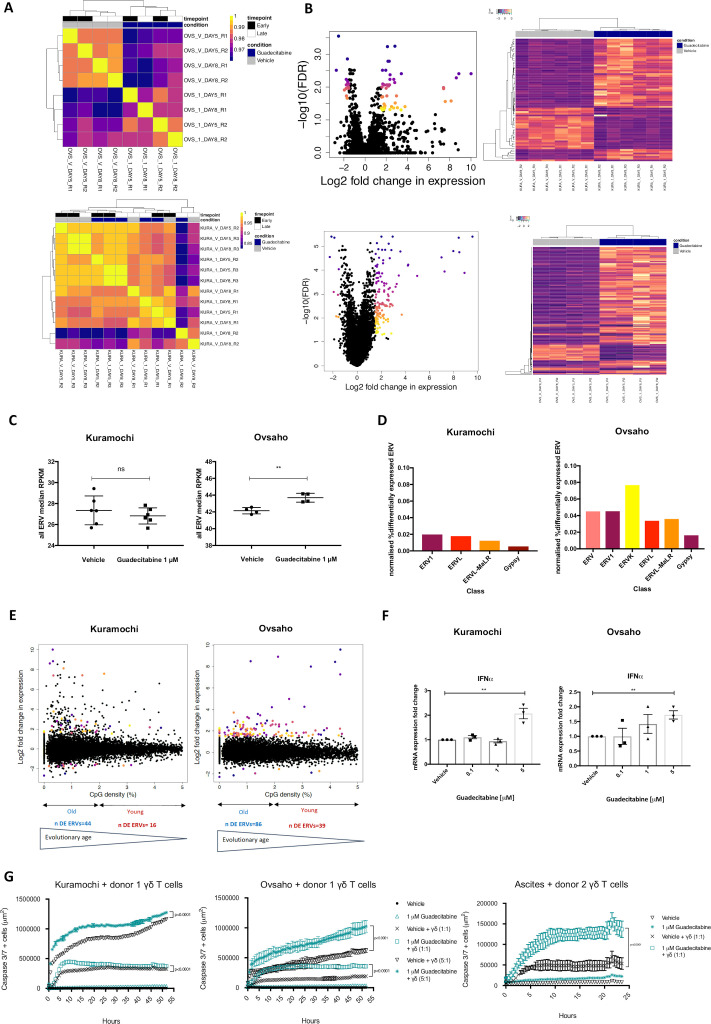
Manipulation of ERV expression threshold by DNMTi increases HGSOC cell lines immunogenicity. (A) Heatmaps of Spearman’s correlation coefficients between guadecitabine and vehicle treated samples from RNA-seq for all ERV repeats analyzed in Kuramochi and Ovsaho. Spearman’s correlation coefficients were calculated between experimental replicates in Kuramochi (A) and Ovsaho (B). Samples are annotated by timepoint and treatment condition. The dendrograms were generated by unsupervised hierarchical clustering and show the relationship between samples. The colors in each sample are indicative of the correlation coefficient and are defined in the color scale. KURA=Kuramochi; OVS=Ovsaho; V=vehicle; 1=1 µM guadecitabine; DAY5=early timepoint; DAY8=late timepoint; R=replicate). (B) Guadecitabine-induced ERV expression changes in HGSOC cell lines; Left (Kuramochi - top and Ovsaho - bottom) Volcano plots showing the log2 fold change in expression against the –log10 FDR adjusted p value for each ERV repeat analyzed at the early and late time points. Significantly DE ERV repeats (absolute logFC>1.5 and FDR adjusted p<0.05) are colored. Right (top and bottom): Heatmaps of DE ERV repeats in guadecitabine-treated Kuramochi and Ovsaho. Each line represents the RPKM values for each DE ERV repeat in each sample from Kuramochi and Ovsaho. Samples are annotated with treatment condition. The dendograms show hierarchical clustering of samples and genes. The colors indicate intensity of expression as annotated in the color scales (top left). (C) Median ERV expression in baseline vs guadecitabine-treated Kuramochi and Ovsaho OC cell lines. Median of RPKM values from all ERV repeats analyzed in each sample for Kuramochi and Ovsaho. The median value for each sample is shown together with the mean±SEM (***p<0.001, t-test). (D) Classification of DE ERVs from guadecitabine-treated Kuramochi and Ovsaho DE ERVs from each cell line’s dataset were assigned to ERV classes according to the annotation database HERVd; the number of DE repeats in each class was normalized to the total number of ERV repeats in each class within the HERVs annotation. (E) Evolutionary age of DE ERV repeats in guadecitabine-treated OC cell lines. LogFC of each DE ERV plotted against its CpG density, normalized by bp size of the element, at the late time point. The DE ERVs were filtered with a percentage CpG density threshold of less than 5. The plot is annotated for evolutionary age, as defined in the work by Ohtani *et al*. CpG densities were calculated using a publicly available annotation of bisulfate sequenced human genome from the Repitools R package. (F) mRNA expression of IFNα in Kuramochi and Ovsaho cell lines treated with increasing doses of guadecitabine. Increasing doses of guadecitabine were used to treat Kuramochi (left) and Ovsaho (right) cell lines before mRNA expression analysis by qPCR. Data is shown as mean±SEM from three biological replicates (**p<0.01, t-test). (G) Real time quantification of Caspase 3/7 expression in guadecitabine-treated HGSOC cell lines or patient ascites-derived primary OC cells in coculture with *ex vivo* activated γδ T cells. Guadecitabine-treated ascites-derived primary OC cells or cell lines were cocultured with γδ T cells from healthy donors at a 1:1 or 5:1 tumor cell to T cell ratio. Apoptosis was quantified in real time using a caspase 3/7 green dye in IncuCyte. Data are presented as mean±SD based on three technical replicates. Statistical differences were analyzed using wilcoxon matched pairs signed ranks test. DE, differentially expressed; DNMTi, DNA methyltransferase inhibitor; ERV, endogenous retrovirus; IFN, interferon; OC, ovarian cancer; qPCR, quantitative PCR.

ERV expression status drove a clear separation between guadecitabine-treated and vehicle-treated Ovsaho cells, but not between 1 µM guadecitabine-treated and vehicle-treated Kuramochi cells (figure 5A). This indicates that only subtle changes in ERV expression may occur in the Kuramochi cell line—which presents higher levels of ERV expression at the baseline—at a 1 µM guadecitabine treatment. Seventy-one ERV repeats were found DE in the guadecitabine-treated Kuramochi samples at either early or late time point, while more than double, 183, in guadecitabine-treated Ovsaho cells (figure 5B). The overall change in ERV expression, measured as the median ERV expression value between all samples, was significant in Ovsaho but not in Kuramochi (figure 5C). This may indicate again that the baseline ERV expression profile dictates the potential for ERV upregulation in response to DNMTi treatment.

On classification of the DE ERVs, there was no specific ERV class that was enriched following guadecitabine treatment and the class representation profile in the DE ERVs differed between the two cell lines. ERVK was the class with the most ERV expression changes in Ovsaho (figure 5D); of note ERVK is one of the evolutionary youngest ERV classes.^17^ Indeed, analysis of the CpG density within each DE ERV sequence revealed that, in the guadecitabine-treated Kuramochi samples, most of the DE changes occurred at CpG densities between 0% and 2% (figure 5E); in the guadecitabine-treated Ovsaho samples the DE changes spread past 2% CpG density (figure 5E). As methylated cytosine within CpG islands are prone to deaminate to thymine over time,^18^ older repetitive elements present less CpG density.^19^

These data indicate that in different cell lines, or distinct ovarian tumors, the mechanisms regulating baseline ERV repression, and therefore their epigenetic-driven re-expression, may vary.

Additionally, we found an increase in IFNα mRNA expression in Ovsaho with increasing doses of guadecitabine. In Kuramochi cells, a significant increase in IFNα mRNA expression was observed only at 5 µM guadecitabine, compared to the vehicle, potentially due to the enhanced ERV and IFN enrichment at the baseline in this cell line (figure 5F).

Next, we cocultured guadecitabine-treated cell lines Kuramochi, Ovsaho or ascitic primary ovarian tumor cells with cytotoxic γδ T cells from healthy donors (1:1 or 5:1 T cell:tumor ratio, using over 85% γδ TCR+ T cells). Using IncuCyte live cell imaging, we measured a significant increase in Caspase 3/7+ cells, when guadecitabine-pretreated Kuramochi and Ovsaho cells were cocultured with γδ T cells at 1:1 or 5:1 T cell:tumor ratio (figure 5G). This effect was similarly reproduced using OC primary cell cultures derived from the ascites of a treatment-naïve patient (figure 5G). Importantly, the significant increase in tumor cell death in the presence of combination guadecitabine and γδ T cell treatment was higher in Kuramochi, compared to Ovsaho.

Altogether these data suggest that distinct baseline ERV expression profiles may significantly influence baseline immunogenicity and efficacy of DNMTi-driven immunomodulation in OC.

## Discussion

Immune infiltration is known to significantly affect patient survival in EOC.^9^ Recent evidence has shown a role for ERV expression in influencing antitumor immunity and consequential immune cell recruitment. In this work, we first investigated the expression of ERVs in OC and their relationship with patient survival and immune infiltration. Using an adapted RNA-seq analysis method, in which a reference ERV annotation is used instead of canonical gene annotation, it was possible to find ERVs expressed in >350 high grade serous EOC samples from TCGA.

The expression of ERVs was found to separate the samples into four main clusters; even though each of these clusters was not associated with a differential OS benefit (data not shown), these data show that different patterns of ERV expression define subgroups of EOC tumors.

In normal cells, epigenetic mechanisms prevent the expression of ERVs.^20^ Altered epigenetic states have been reported in EOC, governing tumorigenesis and driving resistance to platinum-based chemotherapy.^21 22^ The differential ERV expression observed in the TCGA dataset may be a consequence of the different epigenetic landscapes of the tumors.

When looking into each ERV repeat’s association with patient survival, ERVs were found to be associated with both improved or worse OS depending on their genomic location, which may explain why the expression clusters are not associated with survival. This suggests that ERV repeats, belonging to the same family, may affect survival differently, once transcribed, in a yet unknown mechanism. One potential mechanism could be recruitment of transcription factors and regulation of gene expression; indeed, ERV long terminal repeats can act as promoters or enhancers for nearby genes.^23^ Alternatively, the expression of a given ERV at a particular locus may be the result of an either permissive or repressive epigenetic state of the region and therefore a passenger effect of the epigenetic modifications present in their vicinity.

Similarly, a group of ERVs expressed in other cancer datasets from TCGA were shown to have both negative and positive associations with immune signatures,^24^ confirming the dichotomous effects of ERV expression within the same cancer type.

Our analysis has identified the existence of ERV families that are exclusively associated with either a survival advantage or a disadvantage across independent cohorts, robustly indicating that some families of ERVs may specifically affect survival, potentially via their translation into immunogenic ERV antigens. Indeed, some ERVs, particularly evolutionary young ERVs such as HERVK, have retained open-reading frames within their *gag* and *pol* genes^25^ and envelope proteins derived from ERVK have been shown to trigger immune responses in an Indian rhesus macaque model.^26^ Similarly, the existence of ERV-derived immunogenic antigens, capable of triggering adaptive immune responses has been previously demonstrated in renal cancer.^27 28^

Using LASSO logistic regression, it was possible to derive and validate a numerical prognostic score for each TCGA EOC patient in the analysis, based on the expression of 32 ERV repeats, with a high prognostic score being associated with improved prognosis in these patients. Though the prognostic power of the score was more limited in the testing set—particularly for the PFS and potentially due to the reduced number of samples—it strongly validated in a completely independent dataset (HH). Using the TCGA dataset, the ERV prognostic score was strongly positively correlated with the median expression of the 32 ERV features. This indicates that a high expression of these repeats is significantly associated with survival.

The study by Smith *et al*^24^ showed that high average overall ERV expression in a number of tumors (no OC data were included) was associated with worse survival. In our study, unlike the overall median ERV expression, the expression of 32 specific ERVs was found to be positively associated with OS and PFS in EOC. This indicates a qualitative nature, rather than quantitative, of the effect of ERV expression on survival in EOC and potentially other cancers.

Another factor influencing the association between ERVs and survival may be the transcription of the specific ERVs into ERV-derived dsRNAs, able to trigger a RIG-I/MDA5-mediated antiviral response.^7 8^ In our study, the prognostic score was found to positively, although weakly, correlate with the expression of RIG-I, using bioinformatics tools, suggesting that dsRNAs, derived from some or all of the 32 ERV features of the score, may potentially trigger a RIG-I mediated immune response and IFN type I induction. Indeed, a significant positive correlation was also found between the ERV prognostic score and expression of IFNβ. Confirmation of these findings by quantitative PCR (qPCR) in other cohorts is warranted.

BRCA1/2 mutations and CCNE1 amplification are known prognostic factors for OC patients, with BRCA1/2 mutations being predictive of better patient prognosis, while CCNE1 amplification being predictive of worse outcome.^13 15^ It was, therefore, intriguing to find that tumors presenting mutations in BRCA1/2 and those without CCNE1 amplification present a higher ERV score. It has been shown that tumors with defects in DNA repair pathways present a high mutational burden and higher levels of neoantigens.^29 30^ Furthermore, BRCA1/2-mutated HGS ovarian tumors have been shown to exhibit significantly increased CD3+ and CD8+ TILs.^31^ It is possible that the high genomic instability due to defects in BRCA1/2 may determine higher levels of transcription of antigenic ERVs, supporting a link between DNA repair defects, spontaneous expression of ERVs, immunogenicity, and ultimately, survival—though this relationship remains to be further investigated.

Furthermore, we investigated the relationship between ERV expression and immune cell infiltration using computational methods and validating our findings using multiplex IHC. The expression of five non-intragenic ERVs was shown to correlate with that of five known surface markers of activated or exhausted T lymphocytes within EOC tumor tissue. Though this may indicate that these five ERVs could be translated into immunogenic antigens and attract effector T cells to the tumors, confirmation by mass spectroscopy or immunopeptidomics would be ideally used to support this hypothesis further.

Similarly, the ERV score was found to correlate positively, with activated/exhausted T lymphocytes markers PD-1 and LAG3. Importantly, this finding was validated by multiplex IHC in the HH dataset, in which the ERV score correlates with the infiltration of CD8+PD1+ double positive T cells. To our knowledge, this is the first time that an ERV expression signature predicts immune infiltration in ovarian tumors.

In order to further investigate the significance of baseline ERV expression in OC, we compared the transcriptional profiles of EOC cell lines Kuramochi and Ovsaho as well as the ERV transcriptional changes induced by treatment with guadecitabine in the same cell lines, which were chosen as previously defined as best representative of HGSOC from patients.^32^ At the baseline, an enrichment for IFN response genes was found in Kuramochi, compared to Ovsaho, including upregulation of genes for viral response proteins MDA5 and RIG-I, the key actors in recognition of ERV dsRNA during viral mimicry.

A higher level of endogenous expression of MDA5 and RIG-I in Kuramochi may be due to a higher baseline spontaneous transcription of ERV; indeed, when the basal ERV expression profile of the two cell lines was compared, a higher median ERV expression was found in Kuramochi, compared to Ovsaho and, accordingly, there were more ERV repeats upregulated than downregulated in Kuramochi cells, compared to the Ovsaho cell line.

Aberrant baseline expression of ERVs has been shown to occur in cancer cells due to functional inactivation of tumor suppressor proteins, often by loss of DNA methylation, during oncogenesis.^33^ These tumor suppressors are physiologically involved in regulating ERV expression and repression. DNA demethylation using DNMTi has been hypothesized to push ERV expression past a ‘tolerance’ threshold and therefore to enhance of immune responses and therapy.^33^

When the ERV expression profile of the guadecitabine-treated cell lines was examined here, more ERV expression changes, particularly upregulation of ERVs, were found in Ovsaho cells, compared to Kuramochi cells; this may be partly due to the observed higher baseline expression of ERVs in Kuramochi.

Upon DNA demethylation using guadecitabine, an enrichment in IFN response genes was found in the Ovsaho cells. This is in accordance with the hypothesis of a threshold of tolerance of ERV expression, past which an IFN response occurs. Indeed, there was a dose-dependent increase in the expression of IFNα on guadecitabine treatment of Ovsaho cells, compared to the vehicle, as measured by qPCR. In guadecitabine-treated Kuramochi cells, an increase in IFNα was only observed at 5 µM guadecitabine. This indicates a dose dependent effect in that higher doses of DNMTi may induce higher ERV expression and consequential higher IFN type I expression.

The fact that Kuramochi cells were shown to express higher levels of ERVs and IFNα at the baseline, compared to Ovsaho cells, may explain why a differential response could not be measured at lower doses of guadecitabine treatment, by qPCR (ie, at 0.1 and 1 µM doses) and transcriptomics analysis (ie, at 1 µM dose); these doses may only determine subtle changes in ERV and IFNα expression, compared to the baseline. As previously hypothesized,^33^ upon treatment with guadecitabine, there may be an increase in ERV expression, past a ‘tolerance threshold’, which together with changes in gene expression, may push EOC cells towards a more immunogenic profile and higher sensitivity to T cell killing.

Importantly, upon coculture with healthy donor expanded γδ T cells, which are innate-adaptive cytotoxic immune cells, Kuramochi cells with a higher baseline expression of ERVs appeared to be more sensitive to immune killing. In both cell lines, treatment with DNMTi could increase tumor cell death in the presence of γδ T cells.

In keeping with our data, γδ T cell and NK cell ligands MICA, MICB and ULBP1-3 have all been shown to be repressed mainly by histone deacetylation and partly by DNA methylation.^34^ Treatment of cancer cell lines with DNMTi alone or in combination with HDACi resulted in upregulation of MICA and MICB, which resensitized tumor cells to NK cell attack *in vitro*.^35–37^ Furthermore, we and others have previously shown DNMTi-induced upregulation of immunoregulatory genes, including HLA and PD-L1^10 38^; such upregulation is likely another key factor, beside ERV and IFN I induction, governing the observed enhanced immune cell killing of DNMTi-treated tumor cell lines *in vitro*.

A recent report has shown an ‘epigenetic switch’ in the regulation of evolutionary young and old ERVs, defined by their CpG density.^17^ The age of the DE ERVs found in the guadecitabine treatment analysis was assessed here; the majority of the DE ERVs, in both cell lines, presented a percentage CpG density of less than 5, which indicates that low CpG densities are more amenable to hypomethylation by DNMTi and consequential re-expression of associated ERVs. Besides DNA methylation, histone methylation has been demonstrated to regulate ERV repression, particularly of evolutionary ‘old’ ERVs^17^; when classifying the DE ERVs found in the guadecitabine treatment analysis, there was little overlap in ERV class and evolutionary age of the DE elements between Kuramochi cells and Ovsaho cells. This also led to our hypothesis that different cell lines or ovarian tumors may rely on different epigenetic mechanisms of ERV repression. Ohtani *et al* found very little overlap in the numbers and types of ERVs re-expressed following DNMTi treatment of four mixed cancer cell lines,^17^ supporting the hypothesis that the mechanisms governing ERV repression and re-expression may be tumor cell specific.

## Conclusion

In this study, we have shown that an ERV expression signature predicts good prognosis in high-grade serous OC and correlates with immune infiltration of effector T cells in these tumors. Accordingly, we have shown, *in vitro*, that a higher baseline ERV expression may determine higher immunogenicity and dictate the response to DNMTi. Further work may be aimed at using the ERV score to identify those patients which may benefit from manipulation of ERV expression using demethylating agents.

## Methods

### Cell lines, primary ascitic tumor cells and immune cells

Kuramochi and Ovsaho cell lines were purchased from the Japanese Collection of Research Bioresources (JCRB) Cell bank and genetically authenticated by STR profiling conducted by Eurofins Genomics. Cell lines were maintained in RPMI-1640 culture media (Sigma-Aldrich) supplemented with 10% Fetal Bovine Serum (Sigma-Aldrich) and L-glutamine 200 mM, penicillin 10 000 units, streptomycin 10 mg/mL solution (Sigma-Aldrich).

Peripheral blood mononuclear cells (PBMCs) were isolated by Ficoll (Sigma-Aldrich) separation. PBMCs were treated with Recombinant Human Interleukin 2 (IL-2, Peprotech) and 1 µg/mL zoledronic acid (ZA) (Zometa, Novartis) for γδ T cell isolation and supplemented with IL-2 every 48 hours. Human primary immune cells were cultured in RPMI-1640 media (Sigma-Aldrich) with 10% Human AP Serum (Sigma-Aldrich) and L-glutamine 200 mM, penicillin 10 000 units, streptomycin 10 mg/mL solution (Sigma-Aldrich).

Primary EOC cells were isolated from ascites by Ficoll (Sigma-Aldrich) separation and maintained in RPMI-1640 culture media (Sigma-Aldrich) with 20% FBS (Sigma-Aldrich), L-glutamine 200 mM, penicillin 10 000 units, streptomycin 10 mg/mL solution (Sigma-Aldrich), 34 ng/mL insulin (Sigma-Aldrich) and 2.2 mM Sodium Pyruvate (Sigma-Aldrich). Data from the ascites sample used in this study was previously published^10^ and showed that the sample was enriched for cells expressing EOC cell markers WT-1, CA-125 and epithelial cell marker EpCAM, used broadly as a tumor cell marker. All cells were cultured at 37°C with 5% CO_2_.

### Treatment with guadecitabine and co-culture with γδ *T cells*

Guadecitabine was provided by Astex Pharmaceuticals, Inc. and reconstituted in its clinical diluent (65% Propylene Glycol, 25% Glycerin, 10% Dehydrated Ethanol) which was also used as vehicle control. OC cell lines and primary tumor cells were treated with 0.1, 1 and 5 µM guadecitabine or vehicle on day 1 and day 3. Cell culture medium was replaced with fresh medium on day 5. Cell pellets from each condition, to be further processed for RNA-sequencing and qPCR analyses, were taken on day 5 (referred to as early timepoint) or day 8 (referred to as late timepoint). In co-culture experiments, on day 8, tumor cells in each treatment condition were seeded in triplicates onto 96-well plates at a density of 7×103 cells per well and incubated at 37°C with 5% CO2 for 24 hours, before addition of immune cells.

In γδ T cell coculture experiments, 24 hours after seeding of tumor cell lines or primary cells onto 96 well plates (described above), ZA was added to increase isopentelyl-pyrophosphate expression (for higher γδ T cell recognition, as described^39^) on tumor cells and extra wells were kept ZA-untreated as controls. After further 24 hours, γδ T cells were added at various T cell:tumor cell ratios and co-cultured for 24 hours before readout experiments described below. Extra wells were maintained without γδ T cells, as controls.

### IncuCyte live cell imaging

For real-time monitoring of tumor cell killing, γδ T cell co-culture experiments were set up in the presence of 1 µM Green Caspase-3/7 Cell Apoptosis Reagent (Essen Bioscience/Sartorius) and imaged every 45 min using an IncuCyte ZOOM instrument with ×10 magnification for up to 55 hours.

### Quantitative real-time PCR

Total RNA from guadecitabine or vehicle-treated tumor cells was extracted and purified using the RNeasy kit (Qiagen). After quantification of the yield on a Nanodrop instrument, total RNA was converted to cDNA using the High Capacity cDNA Reverse Transcription kit (Applied Biosystems). Real Time PCR was performed using SYBR Green Master Mix (Applied Biosystems) in a 7900HT Real-Time PCR System (Applied Biosystems, Paisley, UK) with standard FAST settings on an SDS 2.4 software (Applied Biosystems) and analyzed using the 2 (-delta delta C(T)) method.^40^ qPCR primers were validated by producing a standard curve with serially diluted (1:4) cDNA inputs. PPIA was used as housekeeping gene. Primer sequences were as follows: *PPIA* Forward: 5’- GTCCTGGCATCTTGTCCATG −3’, *PPIA* Reverse: 5’- CTTGCCATCCAACCACTCAG −3’; *IFNα* Forward: 5′-GACTCCATCTTGGCTGTGA-3′, *IFNα* Reverse: 5′- TGATTTCTGCTCTGACAACCT-3′.

### HH patient cohort

All procedures involving human participants were done in accordance with the ethical standards of the institutional and/or national research committee and with the principles of the 1964 Declaration of Helsinki and its later amendments or comparable ethical standards. 58 EOC patients made up the HH cohort and were treated at the HH, Imperial College London NHS Trust between 2004 and 2019. Data related to part of this cohort was used in a previous study.^41^ Written consent was obtained from all patients included in this study who provided tumor tissue for research. Reporting recommendations for tumor marker criteria were followed throughout this study. Patient demographics, surgical and tumor related data were collected retrospectively from medical records. Staging was defined according to FIGO-criteria for ovarian epithelial carcinoma and optimal debulking was defined by postoperative residual disease <10 mm.

### Immunohistochemistry

IHC was conducted on 2-micron FFPE sections using multi-color immune cell phenotyping for PD-1 (clone NAT 105/E3), CD4 (clone SP35), CD8 (clone SP239) and FOXP3 (clone 346/E7) as previously published^42^ for 47 patient samples from the HH dataset.

We analyzed number of immuno-positive cells/mm^2^ of tissue following independent review of specificity of staining by two scorers (FAM, DP) as described.^43^

### RNA sequencing

Samples for RNA sequencing were taken from Kuramochi and Ovsaho cell lines, treated with 1 µM guadecitabine or vehicle, each at day 5 and day 8 timepoints. Each sample was collected in either two (Ovsaho) or three (Kuramochi) biological replicates. Total RNA was extracted using the RNeasy Plus Mini Kit (Qiagen). RNA samples were then quantified using a Nanodrop machine and RNA integrity was assessed by TapeStation. Only samples with RNA integrity score >8 were used for library preparation. Libraries were prepared using the NEBNext Ultra Directional Library Preparation kit II (NEB), with rRNA depletion, following the manufacturer’s instructions.

Sequencing was conducted on an Illumina HiSeq 2500 instrument with 100 bp, paired end reads, at Imperial College LMS Genomics facility. Around 50–60 million aligned reads were obtained for each replicate.

For HH tissue samples, RNA extraction, library preparation and sequencing were conducted at the Institute of Cancer Research London following standard protocols and using an Illumina NovaSeq 6000 instrument.

Adapter sequences were trimmed by BBDuK (US Dept. of Energy Joint Genomics Institute) and reads were aligned to hg19 using TopHat2. Quality of trimmed reads was assessed using FastQC. A hg19 annotation for human ERVs was obtained from the HERVd database.^44^ Filtered reads were assigned to HERV features using featureCount from the RSubread package allowing reads to be multimapping but with the ‘primary only’ option, which takes primary alignments only into account, similarly as described.^45^ ERVs were filtered by a cut-off of >10 RPKM per ERV in at least two samples. Linear models to identify DE ERVs between samples were generated using the limma package in R. ERVs were considered DE if the absolute log2 fold change in expression was >1.5 and with an FDR adjusted p<0.05. ERVs were annotated into repeats and families using the HERVd as reference.

To generate CpG density plots, the DE ERV logFC values found in the RNA-seq analysis were plotted against the percentage CpG density within each ERV sequence, derived using the Repitools R package.

Gene expression analysis was conducted similarly using the biomaRt package to annotate genes.

### Gene set and pathways enrichment analysis

Gene set and pathway enrichment analysis were performed using genesettest and goana functions from the limma package in R, which use the Wilcox mean rank test on a given statistic, here log fold change values, to test whether a set of genes is highly ranked or enriched relatively to other genes. The Molecular Signatures Database was used to source IFN response gene sets. Further pathway enrichment analysis was run using IPA software237 (QIAGEN), using the pre-calculated RPKM as input. For IPA analysis, the cut-off for DE genes was lowered to an absolute log2 fold change in expression of >0.6.

### Analysis of TCGA transcriptional data and survival analysis

Authorization to download EOC TCGA raw RNA-sequencing data was obtained following an application to the National Cancer Institute Genomic Data Commons (NCI GDC). The GDC Data Transfer Tool Client was used to download 379.bam files on Imperial College High performance computing system, on which the files were analyzed similarly as previously described, to define RPKM values for ERVs within each sample. Matched clinical, mutational and gene expression data was also obtained from the NCI GDC. The ConsensusClusterPlus package in R was used to identify robust clusters of OC patients based on tumor ERV expression, by filtering the 1000 ERVs with the most variable expression across samples and median centering their expression values. We then used the ConsensusClusterPlus to identify robust clusters of OC patients based on tumor ERV expression. OS and PFS were determined using multi-variable Cox proportional hazards adjusting for age, stage, grade, histology and residual disease, using the ERV expression or ERV score as continuous variable. The first quartile of the ERV prognostic score was used as a threshold to define high or low groups in the TCGA and HH cohort. All analyses were performed in R using the survival and survminer packages. Pearson’s product moment correlations between gene expression and ERV prognostic scores were calculated and visualized in R.

### LASSO logistic regression

ERVs that were exclusively associated with OS in TCGA samples with complete clinical data (n=328) were filtered by applying two Cox proportional hazard models, one in which ERV expression values were continuous variables and one in which they were non-continuous. 226 candidate ERVs, exclusively associated with better survival, were obtained and used as input for LASSO) analysis, which performs feature selection by a penalisation system. The LASSO model was built on a training set, made up of 246 randomly selected OC samples from TCGA, using the glmnet package in R with ‘cox’ selected as family and with 10-fold cross-validation. This allowed selection of a Lambda coefficient at which the minimum number of ERV features could be found. 32 ERV features were selected, the weighed sum of which gave a numerical value, named ‘ERV score’. TCGA samples of 82°C were used as testing set. ERV score was similarly calculated using the weighed sum of the ERV features within this set. The ERV prognostic scores were subsequentially used in multi-variable Cox proportional hazards performed as previously described.

Twenty-three out of 32 LASSO-selected ERV features were found expressed in the HH validation dataset (n samples=58) and the ERV score was similarly computed using each feature’s LASSO weight and the expression values (RPKM) within each sample.

### Data visualisation and statistical analysis

Statistical analyses and data visualization were carried out using Prism GraphPad V.5 software, Microsoft Excel and R V.3.6.0. All the packages used in R are listed in online supplemental table S3.

10.1136/jitc-2020-001519.supp10 Supplementary data

## Data Availability

Aligned sequencing data generated for this work are available from the European Genome-phenome Archive (accession number EGAS00001004814). Controlled access to TCGA raw data was obtained by applying to the NIH GDC Data Commons and downloaded using the GDC Data Portal https://portal.gdc.cancer.gov/.
