# Supplementary material for: Transcriptional analysis of multiple ovarian cancer cohorts reveals prognostic and immunomodulatory consequences of ERV expression
Source: J Immunother Cancer. 2021 Jan 12;9(1):e001519. doi: 10.1136/jitc-2020-001519 (PMC7805370; doi:10.1136/jitc-2020-001519)
Supplement: Supplementary data [file jitc-2020-001519supp001.pdf]

Supplementary table and figure legends:

Table S1. ERV expression and overall survival in EOC patients.

Table reporting number of ERV repeats and families the high expression of which is associated with improved or worse OS in TCGA OC samples (top). Venn diagram showing the overlap between ERV families associated with improved and worse OS (bottom).

Figure S1. Consensus clustering analysis of OC TCGA samples (n=378) by ERV expression.

A. Consensus Cumulative Distribution Function (CDF) Plot showing the CDFs of the consensus matrix for each k (indicated by colours), which allows determination of optimal number of sample clusters (k) B. Relative change in area under the CDF curve, confirming optimal number of k as four. C. Heatmap of the consensus matrix for k=4, ordered by the consensus clustering shown as a dendrogram on top of the heatmap. Sample clusters are indicated. The analysis was generated using the ConsensusClusterPlus package in R.

Figure S2. Building an ERV prognostic score using LASSO

Building of ERV prognostic score using least absolute shrinkage and selection operator (LASSO). LASSO was used on a training set made up of 75% of TCGA OC samples.

A. Coefficients were generated for each of the pre-filtered ERV repeats (i.e. features; n=226) and plotted against the calculated LASSO parameter Lambda.

B. Partial likelihood deviance from Cox regression models plotted against each Lambda. Optimal lambda values were obtained using cv.glmnet in R, and indicate optimal g number of features for the model.

Table S2. Components of the ERV prognostic score. ERV ID, ERV family and LASSO weight of the 32 ERV repeats which make up the ERV prognostic score.

Figure S3. Probability of PFS by high or low ERV prognostic score in training and testing datasets.

Progression-free survival of OC patients by high (above first quartile) or low (below first quartile) ERV prognostic score in the training (left; n samples=246) and testing (right; n samples=82) sets from TCGA. The HR was estimated by a multivariable Cox model adjusted for age, stage, grade, histology and residual disease (Log-rank p value). The confidence interval is indicated, in brackets.

Figure S4. Probability of OS by high or low expression of selected ERVs in TCGA dataset.

Progression-free survival of OC patients by high (above median) or low (below median) expression of the five ERVs from Figure 2b in TCGA dataset. The HR was estimated by a multivariable Cox model adjusted for age, stage, grade, histology and residual disease (Log-rank p value). The confidence interval is indicated, in brackets.

Figure S5. Correlations between ERV prognostic score and immune genes in TCGA OC samples. Pearson's product-moment correlations were calculated in R between ERV prognostic score and immune genes *PDCD1*, *LAG3*, *DDX58* and *IFNB1*, in TCGA OC samples (n=328). Linear correlations were plotted in R; correlation coefficients and p values are indicated, as well as the confidence intervals (shaded area).

Figure S6. Dose-dependent LINE-1 methylation changes in DNMTi-treated Kuramochi and Ovsaho HGSOC cell lines.

Methylation of LINE-1 following guadecitabine treatment of Kuramochi and Ovsaho cell lines. Percentage average methylation from four CpG sites within repetitive element LINE-1 in Kuramochi and Ovsaho, 5 and 8 days following first guadecitabine treatment (described in the methods). DNA methylation levels were determined by pyrosequencing and are shown as mean ± SEM based on 3 or 4 biological replicates

(* $p<0.05$; ** $p<0.01$; *** $p<0.001$, t test).

Table S3. Summary of packages and functions used in R 3.6.0

Supplementary methods

DNA Methylation analysis by bisulfite pyrosequencing

Genomic DNA was extracted from tumour cell pellets, collected at the early (day 5) or late (day 8) timepoint of the guadecitabine treatment protocol, using the Qiagen DNA mini kit. DNA samples were quantified using a Nanodrop machine and 500ng total DNA was bisulfite converted using the Zymo Gold methylation kit, together with 0% and 100% methylated control DNA samples. PCR amplification of bisulfite converted DNA was conducted using primers specific to Long Interspersed Nuclear Element-1 (LINE-1), as a surrogate measure of global DNA methylation. One of the primers was biotinylated to allow for following pyrosequencing analysis using a biotinylated product. PCR was conducted using the FastStart Taq DNA Polymerase kit (Sigma-Aldrich) with an initial 6 minutes denaturation 95°C, followed by denaturation for 30 seconds at 95°C, annealing for 30 seconds at 53°C, extension for 30 seconds at 72°C and a final extension for 5 minutes at 72°C. Denaturation, annealing and extension steps were repeated 40 times. PCR products were validated to be specific by 2% agarose gel electrophoresis. Pyrosequencing was then conducted on samples and control bisulfited converted DNA using PyroMarkQ96 technology (Qiagen) according to the manufacturer's instructions. The LINE-1 specific sequencing primers were as follows: Forward 5'-GGATTTTTTGAGTTAGGTGTGGG-3', Reverse 5'-BIOTIN-CAAAAAATCAAAAAATTCCCTTTCC-3', Sequencing 5'-AGGTGTGGGATATAGT-3'.
