# Supplementary figures and images for: Transcriptional analysis of multiple ovarian cancer cohorts reveals prognostic and immunomodulatory consequences of ERV expression
Source: J Immunother Cancer. 2021 Jan 12;9(1):e001519. doi: 10.1136/jitc-2020-001519 (PMC7805370; doi:10.1136/jitc-2020-001519)

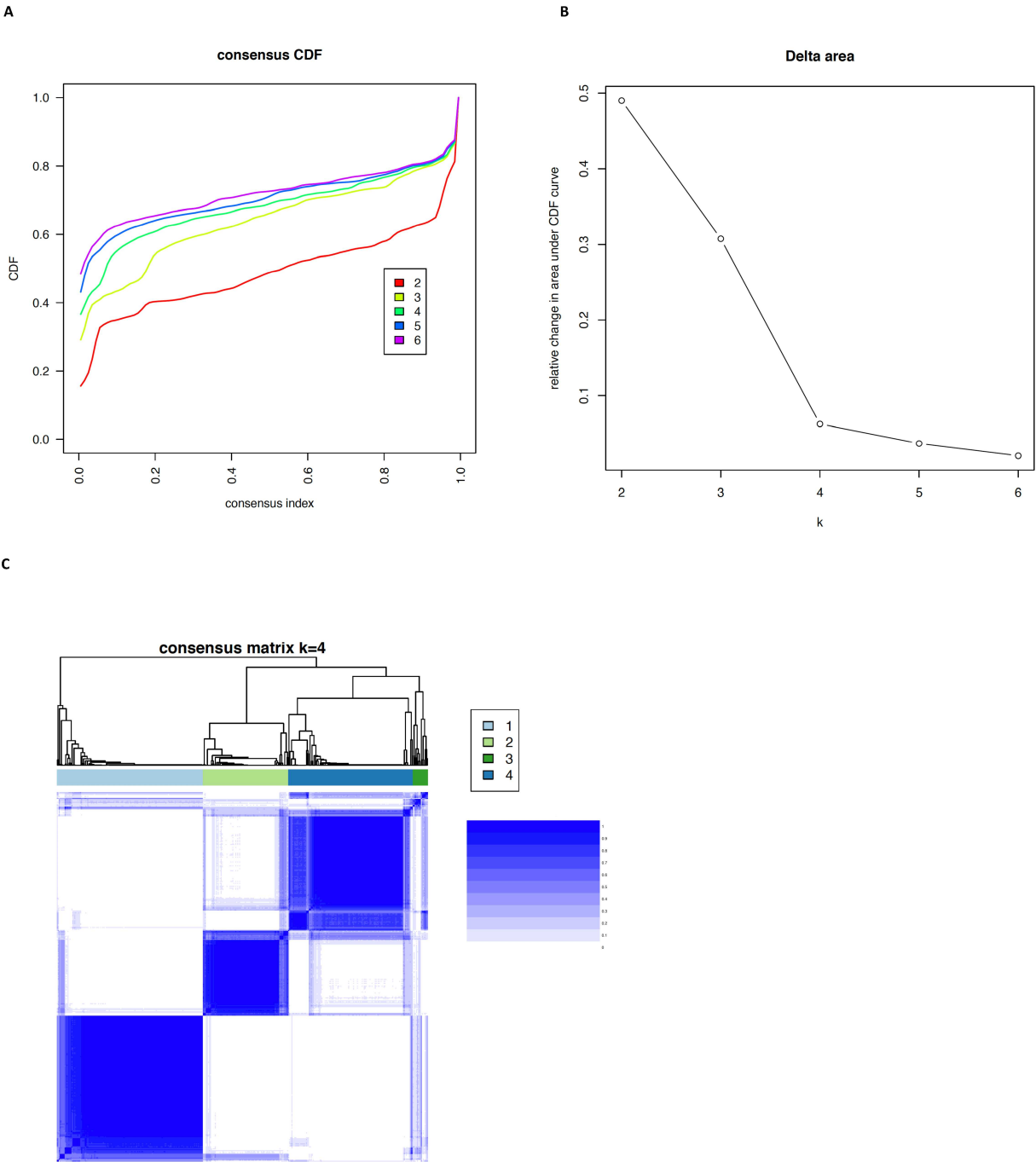

Supplement: Supplementary data [file jitc-2020-001519supp002.pdf]

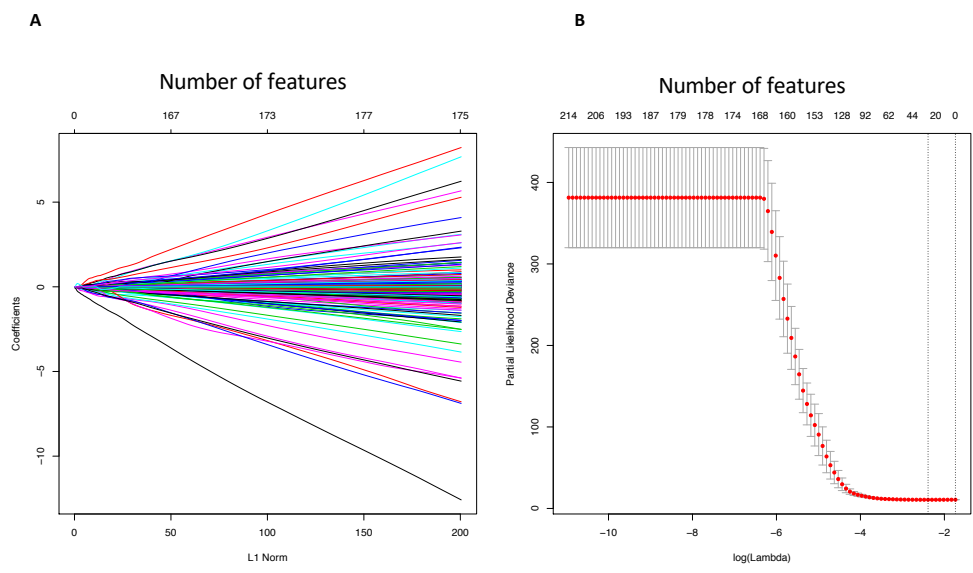

Supplement: Supplementary data [file jitc-2020-001519supp005.pdf]

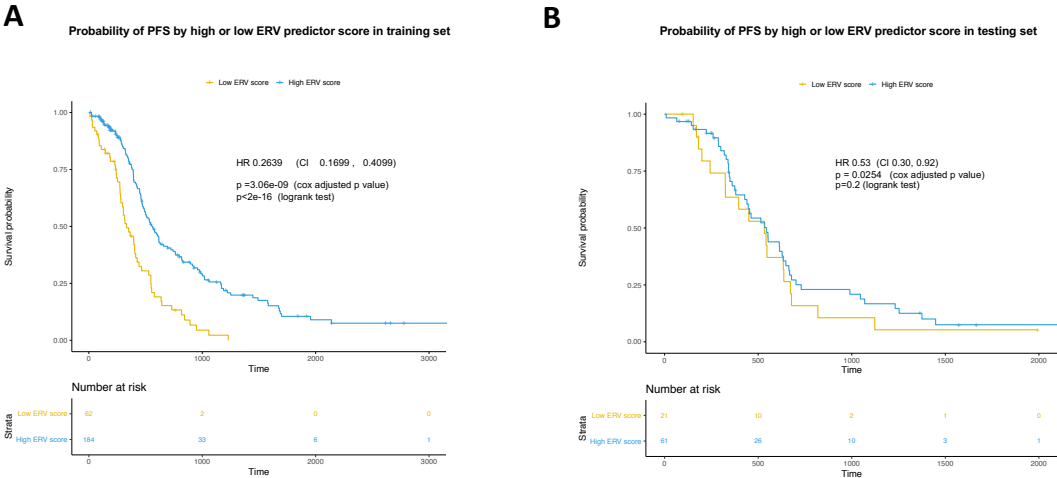

Supplement: Supplementary data [file jitc-2020-001519supp006.pdf]

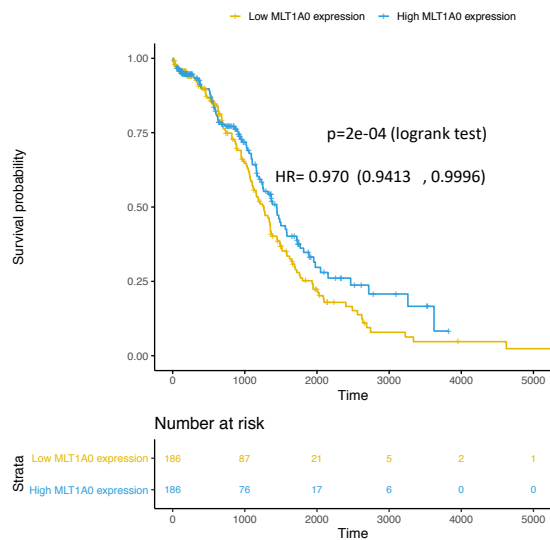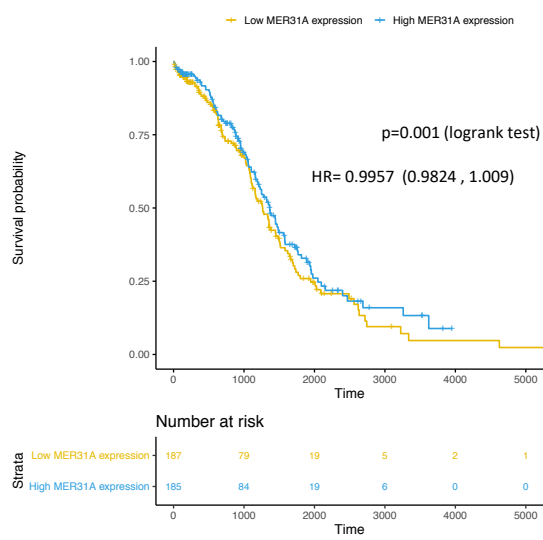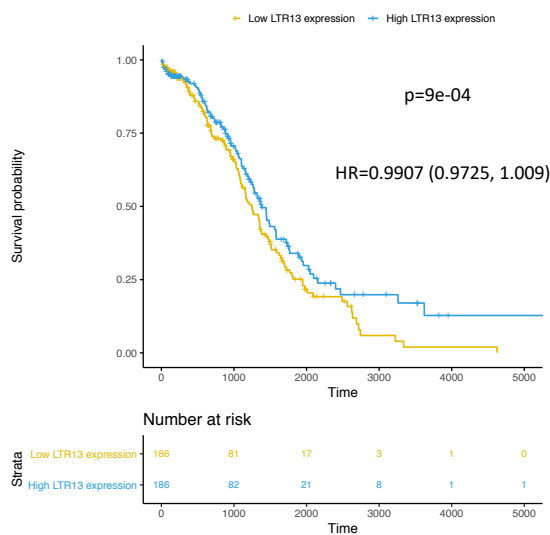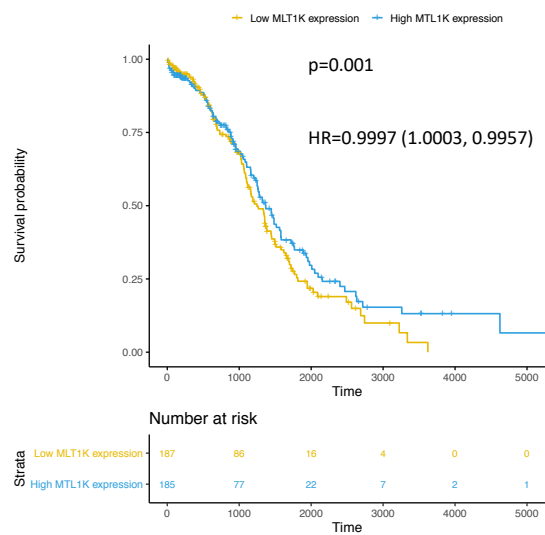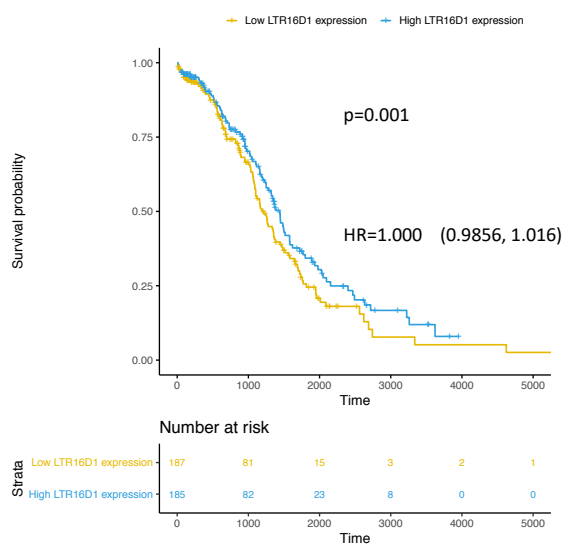

Supplement: Supplementary data [file jitc-2020-001519supp007.pdf]

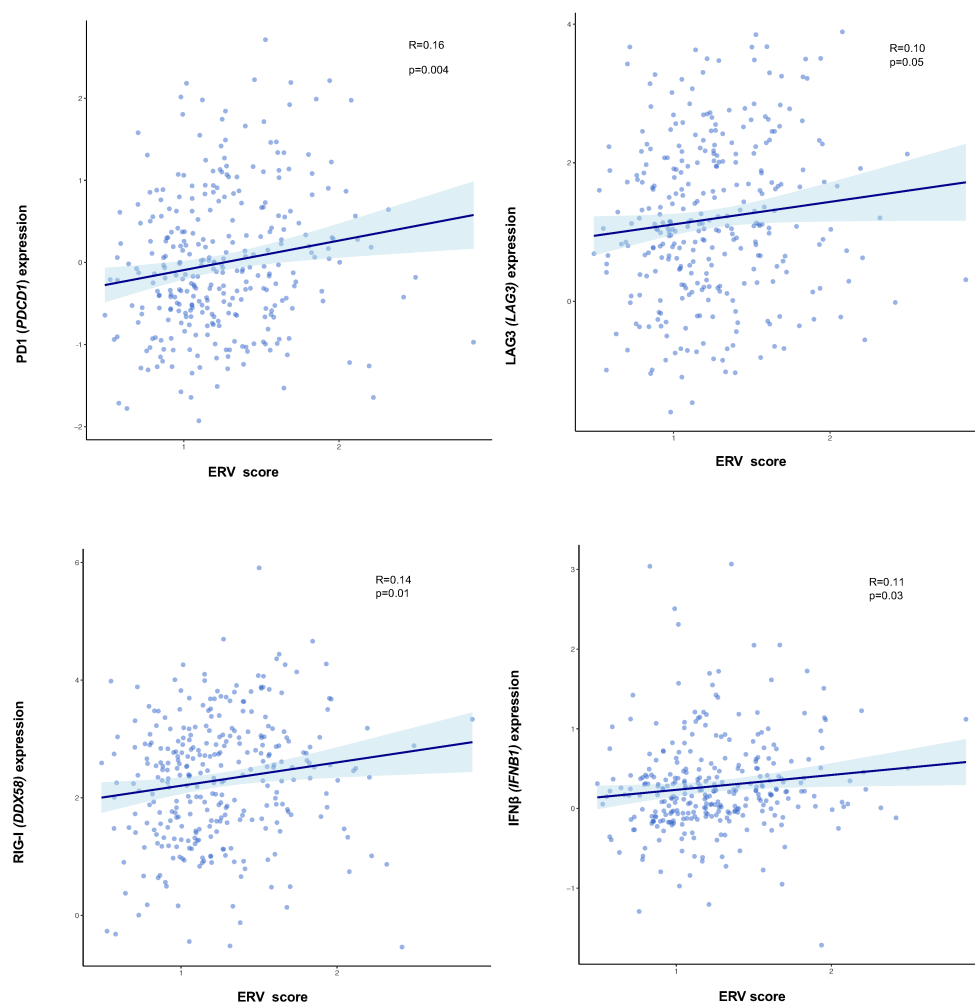

Supplement: Supplementary data [file jitc-2020-001519supp008.pdf]

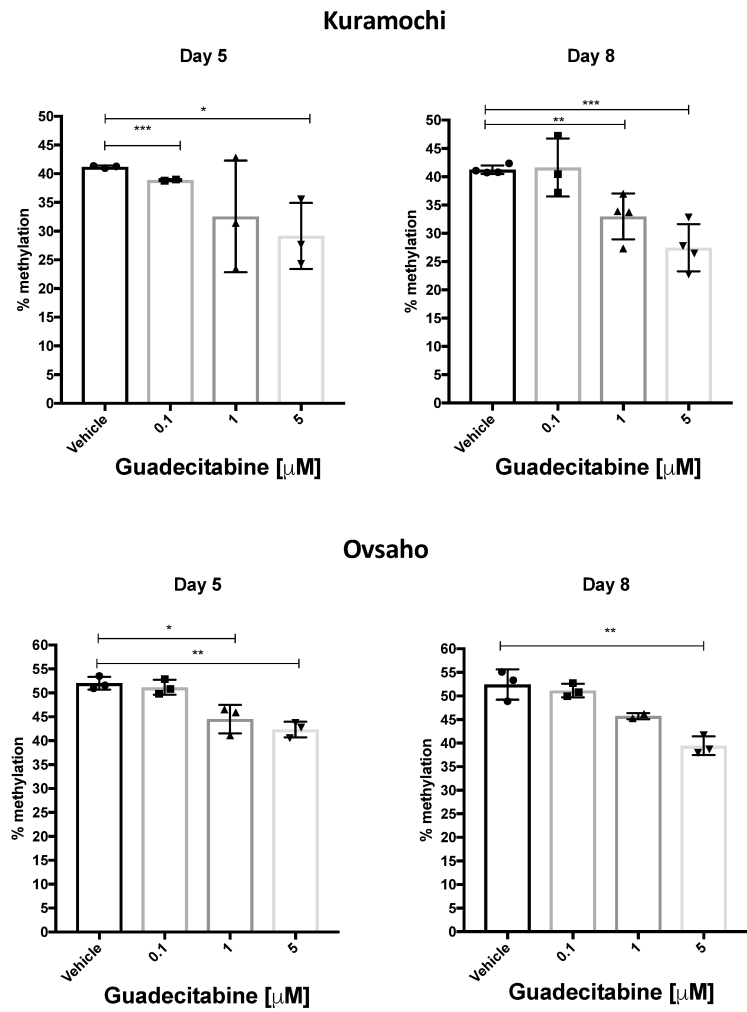

Supplement: Supplementary data [file jitc-2020-001519supp009.pdf]
