# Supplementary material for: Transcriptional analysis of multiple ovarian cancer cohorts reveals prognostic and immunomodulatory consequences of ERV expression
Source: J Immunother Cancer. 2021 Jan 12;9(1):e001519. doi: 10.1136/jitc-2020-001519 (PMC7805370; doi:10.1136/jitc-2020-001519)

Number of ERV repeats analysed	Number of ERV repeats associated with survival advantage	Number of ERV repeats associated with survival disadvantage	Number of ERV families associated with survival advantage	Number of ERV families associated with survival disadvantage	Number of ERV families exclusively associated with survival advantage	Number of ERV families exclusively associated with survival disadvantage
25,207	226	272	115	132	59	76

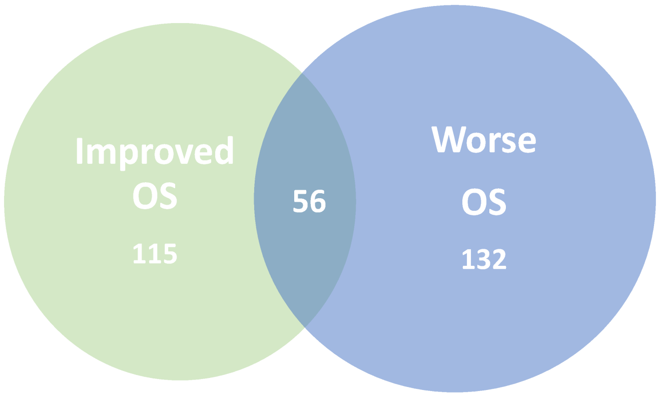

Supplement: Supplementary data [file jitc-2020-001519supp003.pdf]
