# Supplementary material for: Transcriptional analysis of multiple ovarian cancer cohorts reveals prognostic and immunomodulatory consequences of ERV expression
Source: J Immunother Cancer. 2021 Jan 12;9(1):e001519. doi: 10.1136/jitc-2020-001519 (PMC7805370; doi:10.1136/jitc-2020-001519)
Supplement: Supplementary data [file jitc-2020-001519supp004.pdf]

ERV ID	Family	LASSO weight
ERV_0000869	MLT1C	-5.01E-04
ERV_0136673	MLT1N2	-3.01E-03
ERV_0252069	LTR107_Mam	-1.34E-02
ERV_0284225	MER101	-3.74E-02
ERV_0312416	LTR5_Hs	-6.26E-04
ERV_0581642	MLT1J	-4.63E-04
ERV_0587882	PABL_B-int	-7.68E-02
ERV_0703339	LTR41	-5.40E-05
ERV_1468120	MLT1F	-6.48E-03
ERV_1661480	MER4B	-2.90E-02
ERV_1683384	MER21C	-1.03E-03
ERV_1869135	LTR47B3	-1.01E-02
ERV_2070611	MER39	-1.28E-02
ERV_2382813	MER65A	-2.30E-03
ERV_2779506	THE1C	-2.12E-04
ERV_2804251	THE1A	-1.05E-02
ERV_2963122	MLT1F	-4.86E-03
ERV_3035008	MSTA	-1.43E-03
ERV_3087774	MER39	-3.01E-03
ERV_3308062	MLT1J2	-7.05E-05
ERV_3342201	MLT1C	-1.63E-04
ERV_3355129	MLT1H	-2.83E-03
ERV_3357665	THE1B	-2.64E-03
ERV_3480744	LTR78B	-2.59E-03
ERV_3603191	MER4-int	-2.89E-02
ERV_3619438	MER41B	-1.41E-04
ERV_3673831	Harlequin-int	-5.55E-04
ERV_3673833	HERV15-int	-1.71E-04
ERV_3937955	MLT2D	-8.58E-04
ERV_4111570	HERVH-int	-1.46E-02
ERV_4194774	MER4E	-4.72E-03
ERV_4322434	ERVL-E-int	-6.26E-03
