# Supplementary material for: Transcriptional analysis of multiple ovarian cancer cohorts reveals prognostic and immunomodulatory consequences of ERV expression
Source: J Immunother Cancer. 2021 Jan 12;9(1):e001519. doi: 10.1136/jitc-2020-001519 (PMC7805370; doi:10.1136/jitc-2020-001519)
Supplement: Supplementary data [file jitc-2020-001519supp010.pdf]

Function	Package	Source	Purpose
useEnsembl	biomaRt	https://bioconductor.org/packages/release/bioc/html/biomaRt.html	Annotation of gene names
rpkm	edgeR	http://bioconductor.org/packages/release/bioc/html/edgeR.html	Calculation of RPKM values
DGEList	edgeR	http://bioconductor.org/packages/release/bioc/html/edgeR.html	Manipulation of read counts for DE analysis
ggplot	ggplot2	https://cran.r-project.org/web/packages/ggplot2/index.html	Data visualisation
glmnet	glmnet	https://cran.r-project.org/web/packages/glmnet/index.html	LASSO logistic regression
cv.glment	glmnet	https://cran.r-project.org/web/packages/glmnet/index.html	LASSO cross-validation
predict	glmnet	https://cran.r-project.org/web/packages/glmnet/index.html	LASSO predictions
voom	limma	https://bioconductor.org/packages/release/bioc/html/limma.html	Transformation of RNA-seq data for linear modelling
lmFit	limma	https://bioconductor.org/packages/release/bioc/html/limma.html	Fitting linear models for RNA-seq data
eBayes	limma	https://bioconductor.org/packages/release/bioc/html/limma.html	Empirical Bayes statistics for DE analysis
pheatmap	pheatmap	https://cran.r-project.org/web/packages/pheatmap/index.html	Plotting heatmaps
featureCounts	Rsubread	https://bioconductor.org/packages/release/bioc/html/Rsubread.html	Assigning mapped sequencing reads to genomic features
cor/cor.test	stats	https://cran.r-project.org/web/packages/STAT/index.html	Calculating correlation coefficients
survfit	survival	https://cran.r-project.org/web/packages/survival/index.html	Computing an estimate of a survival curve using the Kaplan-Meier method
coxph	survival	https://cran.r-project.org/web/packages/survival/index.html	Computing Cox proportional hazards models
draw.pairwise.venn	VennDiagram	https://cran.r-project.org/web/packages/VennDiagram/index.html	Plotting Venn diagrams
viridis	viridis	https://cran.r-project.org/web/packages/viridis/index.html	Colour palettes
cpgDensityCalc	Repitools	https://bioconductor.org/packages/release/bioc/html/Repitools.html	CpG density calculations
